# Sortilin‐related receptor is a druggable therapeutic target in breast cancer

**DOI:** 10.1002/1878-0261.13106

**Published:** 2021-10-10

**Authors:** Hussein Al‐Akhrass, Mika Pietilä, Johanna Lilja, Ella‐Maria Vesilahti, Johanna M. Anttila, Heidi M. Haikala, Pauliina M. Munne, Juha Klefström, Emilia Peuhu, Johanna Ivaska

**Affiliations:** ^1^ Turku Bioscience Centre University of Turku and Åbo Akademi University Finland; ^2^ Finnish Cancer Institute FICAN South Helsinki University Hospital & Medical Faculty University of Helsinki Finland; ^3^ Institute of Biomedicine, Cancer Research Laboratory FICAN West University of Turku Finland; ^4^ Department of Life Technologies University of Turku Finland; ^5^ InFLAMES Research Flagship Center University of Turku Finland; ^6^ Present address: Janssen‐Cilag Oy Vaisalantie 2 Espoo 02130 Finland

**Keywords:** breast cancer, HER2, HER3, receptor trafficking, SorLA, trastuzumab

## Abstract

In breast cancer, the currently approved anti‐receptor tyrosine‐protein kinase erbB‐2 (HER2) therapies do not fully meet the expected clinical goals due to therapy resistance. Identifying alternative HER2‐related therapeutic targets could offer a means to overcome these resistance mechanisms. We have previously demonstrated that an endosomal sorting protein, sortilin‐related receptor (SorLA), regulates the traffic and signaling of HER2 and HER3, thus promoting resistance to HER2‐targeted therapy in breast cancer. This study aims to assess the feasibility of targeting SorLA using a monoclonal antibody. Our results demonstrate that anti‐SorLA antibody (SorLA ab) alters the resistance of breast cancer cells to HER2 monoclonal antibody trastuzumab *in vitro* and *in ovo*. We found that SorLA ab and trastuzumab combination therapy also inhibits tumor cell proliferation and tumor cell density in a mouse xenograft model of HER2‐positive breast cancer. In addition, SorLA ab inhibits the proliferation of breast cancer patient‐derived explant three‐dimensional cultures. These results provide, for the first time, proof of principle that SorLA is a druggable target in breast cancer.

## Graphical abstract

SorLA interacts with HER2 and HER3 and positively regulates their oncogenic signaling. Here, we report that SorLA‐targeting antibody synergizes with trastuzumab to inhibit proliferation of HER2‐amplified, trastuzumab‐resistant breast cancer cells. In addition, SorLA ab monotherapy inhibits proliferation of breast cancer patient‐derived explant cultures. These results provide proof of principle that SorLA is a druggable target in breast cancer.

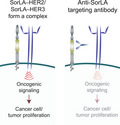

**Abbreviations**
CAM: chick chorioallantoic membrane
CRD: complement‐type repeat domains
EGFR: epidermal growth factor receptor
ERK1/2: mitogen‐activated protein kinase 1 and 2
HER2: human epidermal growth factor receptor 2
HER3: human epidermal growth factor receptor 3
PDECs: patient‐derived explant cultures
SorLA: Sortilin‐related receptor
VPS10P: vacuolar protein sorting 10 protein

## 1 Introduction

The receptor tyrosine‐protein kinase erbB‐2 (HER2) belongs to the HER family of cell‐surface receptors, which transduce extracellular cues into intracellular signals upon receptor homo‐ or heterodimerization [1]. The amplification of the gene encoding HER2 occurs in 15–30% of breast tumors defining a histopathological breast cancer subtype. The diagnosis of HER2‐positive breast tumors guides therapy decisions with anti‐HER2 therapeutics dramatically improving patients’ clinical outcome [1, 2]. However, HER2‐targeted therapies fail in achieving durable efficacy due to acquired resistance leading to distant‐organ metastases in the most challenging clinical setting [3, 4]. Currently, there are no clear treatment options for patients who have progressed after two lines of anti‐HER2 therapy, with reported response rates being extremely poor (ranging from 10% to 22%) [5, 6]. This indicates a highly unmet medical need for patients progressing under current anti‐HER therapies. In the published literature, several therapy resistance mechanisms have been reported, most notably sustained oncogenic signaling that compensates for HER2 inhibition through altered expression of receptor tyrosine kinases such as HER3 [7, 8]. HER2 interacts with HER3 forming the most signaling‐potent dimer among the HER family [9]. Therefore, targeting HER3 may alleviate anti‐HER2 therapy resistance in breast cancer; however, this approach appears to be extremely challenging despite the extensive preclinical and clinical efforts due to HER3 containing a pseudokinase domain in its intracellular region which renders it undruggable with kinase inhibitors [10, 11].

Sortilin‐related receptor (SorLA) is an intracellular sorting protein and a member of the family of vacuolar protein sorting 10 protein (VPS10P)‐domain receptors [12]. The N‐terminal SorLA extracellular/luminal part contains multiple subdomains shown to mediate ligand binding/discharge, while the short C‐terminal tail, containing trafficking signals, binds to cytosolic adaptor proteins to assemble protein complexes orchestrating SorLA traffic [13, 14, 15]. The mature SorLA protein resides mainly in the trans‐Golgi network and undergoes constitutive antero‐ and retrograde trafficking to the plasma membrane through endosomes. Due to its central role in protein trafficking, SorLA has been implicated in the development and/or progression of neurological and metabolic diseases and most recently in cancer [16, 17, 18, 19].

Our previous studies unraveled a HER2 therapy resistance mechanism in which SorLA supports HER2 protein levels and oncogenicity in breast and bladder cancer *in vitro* and *in vivo* [16]. In addition, we have demonstrated that SorLA promotes HER2‐HER3 endosomal recycling to sustain their oncogenic signaling *in vitro* as well as in an anti‐HER2 therapy insensitive brain xenograft *in vivo* model [19]. The present study outlines the translational extension of our previous findings and aims to assess the druggability of SorLA in breast cancer. Our results demonstrate that an anti‐SorLA antibody (SorLA ab) alters the resistance of metastatic breast cancer cells to the HER2 monoclonal antibody trastuzumab *in vitro* and in chick chorioallantoic membrane (CAM) assays. In a HER2‐positive breast cancer mouse xenograft model, SorLA ab and trastuzumab combination treatment inhibits tumor cell proliferation and tumor cell density. In addition, SorLA ab may be clinically relevant since it inhibits the proliferation of patient‐derived HER2‐amplified breast explant cultures. These results demonstrate for the first time that SorLA is an achievable therapeutic target in breast cancer. The implications of this proof‐of‐principle SorLA druggability study may be relevant for other SorLA‐promoted human cancer.

## 2 Materials and methods

Some of the methods employed in this study are the same as those described in our earlier publications [18, 19]

### 2.1 Cell culture and reagents

BT‐474 cells (ATCC, HTB‐20) were grown in RPMI‐1640 (Sigma‐Aldrich, Saint Louis, MO, USA, R5886) supplemented with 10% fetal bovine serum (FBS; Sigma‐Aldrich, F7524), 1% vol/vol penicillin/streptomycin (Sigma‐Aldrich, P0781‐100ML), and l‐glutamine (Sigma‐Aldrich, G7513‐100ML). MDA‐MB‐361 cells (ATCC, HTB‐27) were grown in Dulbecco’s modified essential medium (DMEM; Sigma‐Aldrich, D5769) supplemented with 20% FBS, 1% vol/vol penicillin/streptomycin and l‐glutamine. SK‐BR‐3 cells (ATCC, HTB‐30) were grown in McCoy’s 5A medium, modified with l‐glutamine (Sigma‐Aldrich, M9309) supplemented with 10% fetal bovine serum and 1% vol/vol penicillin/streptomycin. All cells were cultured in a humidified incubator set at 5% CO_2_ and 37 °C. All cells were tested bimonthly for mycoplasma using MycoAlert^TM^ mycoplasma detection kit (Lonza, #LT07‐418) and MycoAlert^TM^ assay control set (#LT07‐518). Cell lines were not independently authenticated for this study. The antibodies used are described in Table S1.

### 2.2 Western blot

Cells were washed with ice‐cold Dulbecco's phosphate‐buffered saline (DPBS, Gibco™, 11590476) prior to lysis with cell lysis buffer (CST, #9803) supplemented with 1% protease/phosphatase inhibitor cocktail (CST, #5872). Lysates from the MMTV/C‐neu transgenic mice [20] were generously provided by Jukka Westermarck (Turku Bioscience Centre, [21]). Cell lysates were sonicated and cleared by centrifugation at 18 000 ***g*** for 10 min. 30 µg of cleared lysates was subjected to SDS/PAGE under denaturing conditions (4–20% Mini‐PROTEAN TGX Gels) and were transferred to nitrocellulose membranes (Bio‐Rad Laboratories). Membranes were blocked with Odyssey blocking buffer (LI‐COR Biosciences, #927‐40000) and incubated with the indicated primary antibodies, diluted in blocking buffer and PBS, overnight at +4 °C. Membranes were then washed three times with TBST (Tris‐buffered saline and 0.1% Tween 20) and incubated with fluorophore‐conjugated secondary antibodies diluted (1 : 2000) in blocking buffer at room temperature for 1 h. Membranes were scanned using an infrared imaging system (Odyssey; LI‐COR Biosciences). The following secondary antibodies were used: donkey anti‐mouse IRDye 800CW (LI‐COR, 926‐32212), donkey anti‐mouse IRDye 680RD (LI‐COR, 926‐68072), donkey anti‐rabbit IRDye 800CW (LI‐COR, 926‐32213), and donkey anti‐rabbit IRDye 680RD (LI‐COR, 926‐68073). The band intensity of each target was quantified using ImageJ (NIH) [22] and normalized to the loading control band intensity in each lane.

### 2.3 Transient siRNA‐mediated knock down

Cells were transfected 72 h before experiments using Lipofectamine RNAiMAX reagent (Invitrogen, Waltham, MA, USA, P/N 56532) according to the manufacturer’s instructions. SORL1‐targeting siRNAs were obtained from Dharmacon—siSORL1 #3 (J‐004722‐07, (5’CCGAAGAGCUUGACUACUU3’)), siSORLA #4 (J‐004722‐05, (5’CCACGUGUCUGCCCAAUUA3’)). Allstars (Qiagen, Hilden, Germany, 1027281) was used as a negative control. All siRNAs were used at a final concentration of 20 nm.

### 2.4 Cell viability assays

Cells were silenced for SorLA in 6‐well plates and then replated on 96‐well plates (5000 cells·well^−1^) in a volume of 100 µL and allowed to grow for 72 h. After experiments, 10 µL·well^−1^ of WST‐8 (cell counting kit 8, Sigma‐Aldrich, 96992) reagent was added. After 3 h of incubation at 37 °C, 5% CO2, absorbance was read at 450 nm (Thermo, Multiscan Ascent). Medium without cells was used as a background control, subtracting this from the sample absorbance readings. Cell viability was calculated as a ratio of endpoint absorbance relative to control cells.

### 2.5 Soft‐agar assay

Bottom agar (1.2%, prepared in normal medium) was casted at the bottom of 12‐well plates. BT‐474 cells stably expressing green fluorescent protein (GFP) with either control shRNA or SORL1 shRNA were resuspended at a concentration of 20 000 cells/1.5 mL in agar (top agar; 0.4% prepared in normal medium) and seeded on top of the pre‐casted bottom agar. Plates were shortly incubated at + 4 °C to accelerate the solidification of top agar, thus maintaining the single‐cell suspension and preventing cells from dropping down to the border between the top and bottom agar layers. After top agar was solidified, 1 mL of normal medium was added on top. Medium was changed once a week. Soft‐agar colony size was measured after 5 weeks of growth by imaging the GFP signal on a fluorescence microscope and measuring the area of GFP‐positive colonies using Image J (NIH) [22].

### 2.6 Colony formation assay

MDA‐MB‐361 and BT‐474 expressing either control shRNA or SORL1 shRNA were seeded on 6‐well plates (1000 cells·well^−1^). Medium was changed twice a week and after 4 weeks of growth colony number was assessed by manual counting of colonies with more than 10 cells.

### 2.7 Cell cycle analysis

BT‐474 cells transfected with control or SorLA‐targeting siRNA were trypsinized, harvested using centrifugation, and suspended in ice‐cold PBS (1 mL). Cells were fixed with 70% EtOH (ice cold) added in a dropwise manner with gentle vortexing of cells. Cells were then stored at + 4 °C until propidium iodide (PI; Sigma‐Aldrich, #P4864‐10 mL) staining was performed. Cells were washed twice with PBS, suspended in PI solution (25 µg·mL^−1^ PI solution in PBS with RNAase A; MACHEREY‐NAGEL, #740505), and incubated for 10 min on ice. Samples were protected from light and kept on ice until flow cytometry analysis. Samples were run on an LSR II flow cytometer, and cell cycle analysis was performed with FlowJo (BD Biosciences, Franklin Lakes, NJ, USA).

### 2.8 Chorioallantoic membrane (CAM) assay

Fertilized chicken eggs were washed with 70% EtOH, and the development was started by placing the eggs in a 37 °C incubator with 50–60% moisture (egg developmental day 0 (EDD0)). On EDD3, a small hole was made in the eggshell to drop the CAM. On EDD10, the eggshell was opened and a plastic ring was placed on the CAM and one million control‐ or SORL1 siRNA‐transfected cells were implanted inside the ring in 20 μL of 50% Matrigel. On EDD16, tumors were imaged, dissected, and weighed.

For CAM treatment experiments, the eggshell was opened and a plastic ring was placed on the CAM on EDD7. One million MDA‐MB‐361 cells, with either IgG control, anti‐SorLA antibody, trastuzumab, or trastuzumab in combination with anti‐SorLA antibody (customized without azide, and with low endotoxin levels), were implanted inside the ring in 20 μL of 50% Matrigel. On EDD10, the antibody treatments were repeated by pipetting antibody solutions on tumors inside the ring. The ring, which contains the tumor, was cut and tumors were dissected and weighed on EDD12.

### 2.9 Orthotopic in vivo xenografts

For orthotopic inoculation, MDA‐MB‐361 cells were suspended in PBS with 50% Matrigel. Cells (5 × 10^6^ in 50 μL) were injected orthotopically into the abdominal mammary gland fat pad of 7‐ to 8‐week‐old ovariectomized Nude female mice (Hsd : Athymic Nude‐foxn1nu, Envigo, France) under isoflurane anesthesia. During the same anesthesia, a 60‐day release E2 (1.5 µg·day^−1^) MedRod implant (PreclinApps Ltd) was also inserted s.c. at the flank of the mice for estrogen supplementation. Therapies were administrated (i.p.) twice a week from day 15 post‐engraftment when the average size of tumors was palpable and had reached 100 mm^3^. Mice were blindly randomized into three groups: a control group receiving a combination of lgG1 (1 mg·kg^−1^) and lgG2a (10 mg·kg^−1^), a monotreatment group receiving trastuzumab (1 mg·kg^−1^), or a combination treatment group receiving trastuzumab (1 mg·kg^−1^) and anti‐SorLA antibody (10 mg·kg^−1^). Mice were monitored daily for any signs of distress, and tumors were measured every 3‐4 days. Tumor volume was calculated according to the formula [tumor volume = (L × W × W) × 0.5], where “L” is the length and “W” is the width of xenograft. Mice were sacrificed at day 35 post‐engraftment, and tumors were dissected, fixed in 10% formalin, and processed for paraffin sections with standard protocols. Sections were stained for the proliferation marker Ki‐67. Mice were housed in standard conditions (12‐h light/dark cycle) with food and water available ad libitum. All animal studies were ethically performed and authorized by the National Animal Experiment Board and in accordance with The Finnish Act on Animal Experimentation (animal license number ESAVI‐9339‐04.10.07‐2016).

### 2.10 Immunohistochemistry analysis of mouse xenografts

Formalin‐fixed, paraffin‐embedded tissue samples were cut into 4‐μm sections, deparaffinized, and rehydrated with standard procedures. For immunohistochemistry (IHC) of tumors, heat‐mediated antigen retrieval was done in citrate buffer (pH 9). Sections were washed with a 0.05 m Tris/HCl pH 7.6, 0.05% Tween 20 washing buffer, blocked for endogenous hydrogen peroxide activity, and incubated with normal antibody diluent (NABD; Immunologic, #BD09‐125). Sections were then incubated with a Ki‐67 antibody (Millipore, #AB9260; diluted 1 : 1000) for 1 h. After washes, samples were incubated for 30 min with a BrightVision goat anti‐rabbit HRP (Immunologic #DPVR110HRP) secondary antibody and washed again. After washes, DAB solution (DAKO, #K3468) was added for 10 s followed by washing. After counterstain with Mayer’s HTX, slides were dehydrated, cleared in xylene, and mounted in Pertex. Stained samples were imaged with Pannoramic P1000 Slide Scanner (3DHISTECH Ltd) and analyzed with QuantCenter software with NuclearQuant quantification module (3DHISTECH Ltd).

### 2.11 Immunohistochemistry staining of patient‐derived primary explant cultures

Fresh tissue was obtained from the elective breast cancer surgeries performed at the Helsinki University Central Hospital (Ethical permit: 243/13/03/02/2013/ TMK02 157 and HUS/2697/2019 approved by the Helsinki University Hospital Ethical Committee). Patients participated in the study by signing an informed consent following the Declaration of Helsinki principles.

Tissues were fixed with 4% paraformaldehyde (PFA) and embedded in paraffin. The samples were sectioned in 5‐μm slices and deparaffinized. The heat‐induced antigen retrieval was performed with a microwave oven in a citrate buffer solution pH9 (Dako). Histochemical staining was carried out using standard techniques for IHC with 1 : 300 antibody concentration. Images were taken with a Leica DM LB microscope (Biomedicum Imaging Unit, University of Helsinki).

### 2.12 Patient‐derived breast cancer explant cultures (PDECs)

Patient tumor samples were collected from consenting patients with permission, approved by the Hospital District of Helsinki and Uusimaa (Ethical permit: 243/13/03/02/2013). A small sample was cut out from a primary tumor obtained from breast cancer surgery and transferred to the laboratory. Samples were minced with a blade and incubated overnight with gentle shaking (130 r.p.m.) at + 37 °C in Mammocult basal medium (StemCell Technologies) containing 0.2% Collagenase A (Sigma), Mammocult proliferation supplements (StemCell Technologies), 4 μg·mL^−1^ heparin, and 50 μg·mL^−1^ gentamicin and penicillin/streptomycin (all from Sigma). On the following day, the mixture was centrifuged at 353rcf for 3 min and the pellet was resuspended in 1 mL of Mammocult basal medium. Fragments were recentrifuged, resuspended in Matrigel (BD), and seeded in 8‐well chamber slides (Thermo Scientific) for 3D culture. Samples were cultured in the Mammocult medium described above.

### 2.13 Immunofluorescence (IF) staining

3D cultured patient samples were fixed with 4% PFA, permeabilized with 0.25% Triton X‐100, and blocked with 10% goat serum in IF buffer (0.1% BSA, 0.2% Triton X‐100, 7.7 mm NaN^3^, and 0.05% Tween 20 in PBS). Primary antibodies were diluted in blocking buffer and 3D cultures were stained overnight. After three washes with IF buffer, samples were incubated with secondary antibodies in blocking buffer and washed again. Nuclei were counterstained with DAPI (Thermo Fisher Scientific, Waltham, MA, USA) and mounted with Immu‐Mount reagent (Thermo Fisher Scientific). Imaging was performed using a Leica TCS CARS SP8 confocal microscope (Biomedicum Imaging Unit, University of Helsinki).

### 2.14 Statistical analyses

At least three independent biological replicates were performed for each experiment. The sample size (*N*) and the related statistical methods are described within figure legends. When data deviated from a normal distribution based on the D’Agostino‐Pearson normality test, non‐parametric statistical tests were used. Significance was concluded when a probability value (*P*‐value) was lower than 0.05. NS, not significant; *P < 0.05; **P < 0.01; ***P < 0.001; ****P < 0.0001. In every case, unequal variances between groups of data were assumed and two‐tailed *P*‐values were reported. No power analyses were conducted to estimate sample size.

## 3 Results

### 3.1 Targeting HER2 downregulates SorLA levels

**Fig. 1:**
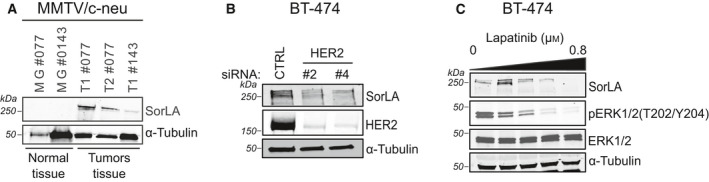
HER2 targeting decreases SorLA levels. (A) HER2‐driven tumorigenesis increases SorLA levels. Representative immunoblotting of SorLA, with α‐tubulin as a loading control, from normal mammary glands (MG#077 and MG#0143) and breast tumors (T1#077, T2#077, and T1#143) from the MMTV/c‐neu transgenic mice [20]. T1#077 and T2#077 represent different tumors from the same mouse. (B) HER2 silencing decreases SorLA levels. Representative immunoblotting of SorLA and HER2, with α‐tubulin as a loading control, from HER2‐silenced BT‐474 cells. (C) Lapatinib decreases SorLA levels. BT‐474 cells were treated with increasing concentrations of lapatinib (0, 0.1, 0.2, 0.4, and 0.8 µm) for 24 h. Representative immunoblotting of SorLA, pERK1/2(T202/Y204), and total ERK1/2, with α‐tubulin as a loading control.

SorLA promotes HER2 and HER3 oncogenic signaling and exhibits increased expression in brain‐trophic metastatic breast cancer cells [18, 19]. Whether HER2‐induced tumorigenesis alters SorLA levels remains unknown. We used the MMTV/C‐neu transgenic mice, a canonical mouse model of HER2‐positive breast vcancer [20], to compare SorLA levels between healthy mammary glands and breast tumors. We found a heterogeneous increase in SorLA levels in breast tumors (Fig. 1A), indicating that HER2‐mediated cell transformation increases SorLA expression. Accordingly, HER2 silencing with two independent siRNAs decreased SorLA levels in human HER2‐positive (HER2+) BT‐474 breast cancer cells (Fig. 1B). As an additional control, we targeted HER2 signaling using the dual EGFR/HER2 tyrosine kinase inhibitor lapatinib and assessed SorLA levels in the lapatinib‐sensitive HER2+ BT‐474 and SK‐BR‐3 breast cancer cells. Lapatinib treatment was efficient as demonstrated by inhibition of the phosphorylation of mitogen‐activated protein kinase 1 and 2 (ERK1/2) (Fig. 1C and Fig. S1). In line with the siRNA‐mediated depletion of HER2, we found that HER2 inhibition with lapatinib decreases SorLA levels in BT‐474 and SK‐BR‐3 cells (Fig. 1C and Fig. S1). HER2 monoclonal antibody trastuzumab did not alter SorLA levels (Fig. S1B–E), indicating that modulating SorLA levels downstream of HER2 requires HER2 inhibition using a tyrosine kinase inhibitor. Altogether, these results demonstrate that HER2 expression and kinase activity positively regulate SorLA in breast cancer.

### 3.2 SorLA silencing blocks S‐phase entry and inhibits clonogenic growth

**Fig. 2:**
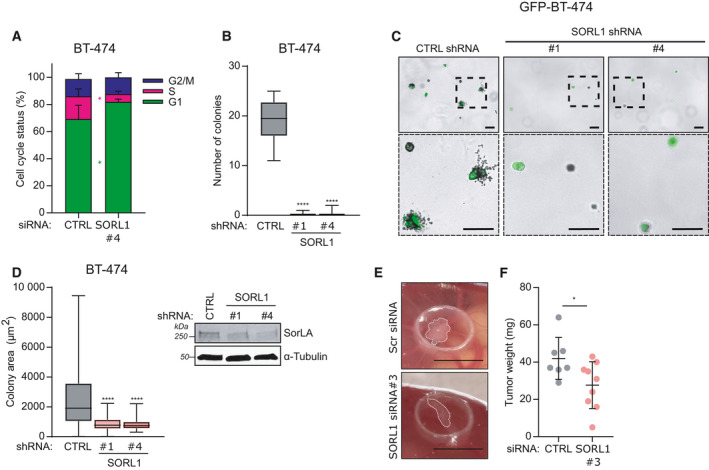
SorLA silencing blocks the cell cycle and inhibits clonogenic growth. (A) SorLA silencing inhibits cell cycle S‐phase entry. BT‐474 cells were silenced for SorLA expression and cells were labeled using the DNA dye propidium iodide (PI) prior to analysis by flow cytometry. Results are represented as mean ± SD, *n* = 3. (B) SorLA silencing inhibits colony formation. Colony formation assay using BT‐474 cells stably expressing CTRL shRNA, SORL1 shRNA#1, and shRNA#4. Results are represented as median ± min to max, *n* = 3. (C) SorLA silencing inhibits colony formation in soft agar. Representative images of soft‐agar colony formation assay with GFP‐expressing CTRL shRNA, SORL1 ShRNA#1, and shRNA#4 BT‐474 cells. Scale bars: 100 µm. (D) Quantification of colony area from samples described in (C). Results are represented as median ± min to max. *n* = 3 in total ≥ 20 spheroids/group. A representative western blot validating SorLA silencing is shown. (E) SorLA silencing inhibits *in ovo* tumor growth. *In ovo* chorioallantoic membrane (CAM) tumor formation assay with SorLA‐silenced MDA‐MB‐361 cells. Scale bars 7 mm. (F) Tumors described in (E) were weighed, and the results are represented as mean ± SD, siCTRL 7 tumors, siSORL1#3 9 tumors. Statistical analyses: *P < 0.05; ****P < 0.0001 A, Student’s *t*‐test (unpaired, two‐tailed, unequal variance); B, Kruskal–Wallis, Dunn’s multiple comparisons test; D, one‐way ANOVA, Dunnett’s multiple comparisons test; F, Mann–Whitney *U*.

Using an array of breast cancer cell lines exhibiting low, moderate, and high endogenous SorLA levels, we have previously demonstrated that SorLA regulates cell viability [18, 19]. This is reconfirmed in this study using BT‐474 and MDA‐MB‐361 cells (Fig. S2A and S2B). The effect of SorLA silencing, however, on cell cycle progression remains unknown. Cell cycle analysis using flow cytometry revealed that SorLA silencing triggers an increase and a decrease in the percentage of cells in G1‐ and S‐phase, respectively (Fig. 2A). This indicates that SorLA depletion results in a blockade of the cell cycle at G1/S transition, in line with the decreased cyclin D1 expression in SorLA‐depleted cells [18]. Next, we used our previously established model of breast cancer cells expressing a specific and validated *SORL1* shRNA [18] to investigate the role of SorLA in clonogenic growth. SorLA silencing abolished the ability of single BT‐474 and MDA‐MB‐361 cells to grow into colonies (Fig. 2B and Fig. S2C). Furthermore, we investigated the effect of SorLA silencing in anchorage‐independent growth using the soft‐agar colony formation assay. In line with the results of the clonogenic assay, we found that SorLA silencing inhibits colony formation in soft‐agar (Fig. 2C and 2D). To validate the relevance of targeting SorLA in tumors, we set up CAM assays using HER2 therapy‐resistant and sensitive MDA‐MB‐361 and BT‐474 cells, respectively [24]. Silencing SorLA inhibited tumor growth of both cell lines on the CAM (Fig. 2E and 2F; Fig. S2D and S2E). Cumulatively, these results demonstrate a role of SorLA in promoting cell cycle progression, cancer cell clonogenicity, and tumor growth in HER2‐positive breast cancer cells. We aimed next to assess the druggability of SorLA in breast cancer.

### 3.3 SorLA ab counteracts resistance to HER2 monoclonal antibody trastuzumab

**Fig. 3:**
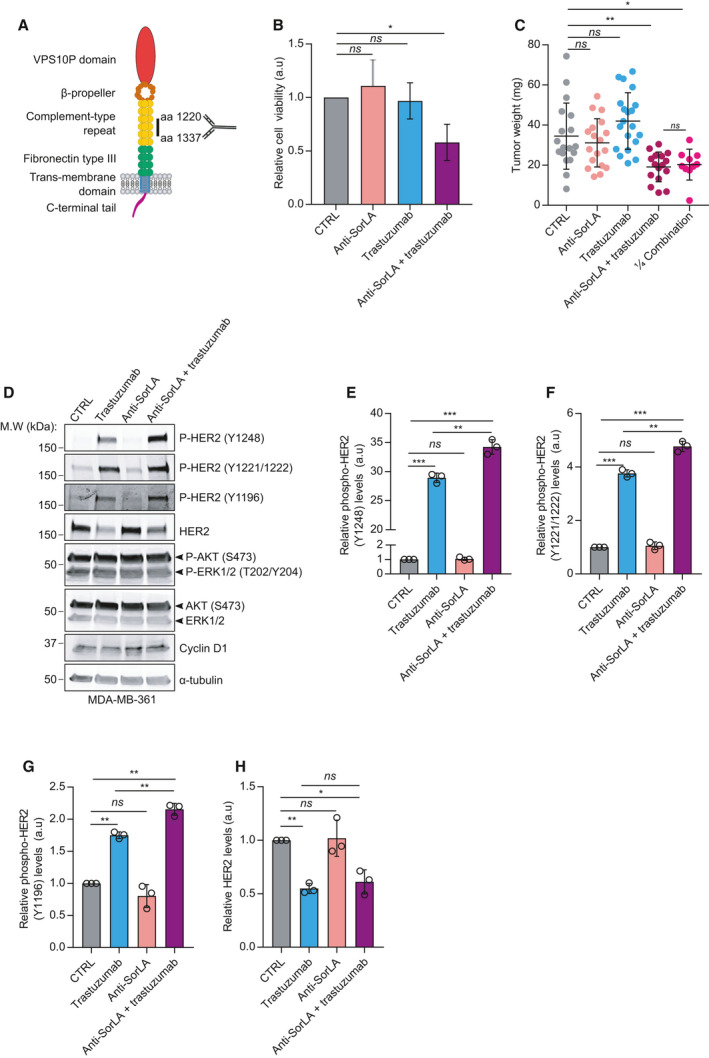
Anti‐SorLA antibody alters trastuzumab resistance *in vitro* and *in ovo*. (A) Representative scheme of SorLA domains. The epitope in the complement‐type repeat domains targeted by anti‐SorLA antibody is shown. (B) Anti‐SorLA antibody synergizes with trastuzumab *in vitro*. MDA‐MB‐361 cells were treated for 3 days with either IgG control (20 µg·mL^−1^), anti‐SorLA antibody (20 µg·mL^−1^), trastuzumab (10 µg·mL^−1^), or a combination of anti‐SorLA and trastuzumab. Cell viability was assessed using the WTS‐8‐based method. Results are represented as mean ± SD. Synergism was assessed using two‐way ANOVA, *P = *0.0381, *n* = 3. (C) Anti‐SorLA synergizes with trastuzumab altering the resistance of MDA‐MB‐361 tumors *in ovo*. MDA‐MB‐361 cells were engrafted *in ovo* (CAM assay) and treated at days 0 and 3 with 9 µg/10^6^ cells either IgG control, anti‐SorLA antibody, trastuzumab, or a combination of anti‐SorLA and trastuzumab in a volume of 20 µL (final treatment concentration = 450 µg·mL^−1^) and tumors were imaged and weighed at day 5 post‐engraftment. ¼ combination corresponds to a 75% reduced concentration of anti‐SorLA + trastuzumab (reduced treatment concentration = 112.5 µg·mL^−1^). Synergism was assessed using two‐way ANOVA; *P = *0.0024, 18 tumors from 2 independent experiments/group. (D) Trastuzumab‐resistant MDA‐MB‐361 cells were treated with either IgG control (20 µg·mL^−1^), trastuzumab (10 µg·mL^−1^), SorLA ab (10 µg·mL^−1^), or a combination of trastuzumab and SorLA ab (20 µg·mL^−1^) for 48 h prior to western blot analyses of whole‐cell lysates using the indicated antibodies. (E‐G) Band intensity quantification of HER2 phosphorylation (from experiments described in D) normalized to total HER2 and relative to IgG‐treated control sample. (H) Band intensity quantification of HER2 levels normalized to loading control and relative to IgG‐treated control sample. Results are represented as mean ± SD, *n* = 3. Statistical analyses: *P < 0.05; **P < 0.005; ***P < 0.001, one‐way ANOVA, Dunnett’s multiple comparisons test unless otherwise indicated.

SorLA is mainly localized at the trans‐Golgi network and undergoes continuous endosomal trafficking between the Golgi and the cell surface [12]. Furthermore, we have established that SorLA extracellular domain interacts directly with HER2 and HER3 and that this interaction is necessary for SorLA‐dependent proliferation of HER2‐positive breast cancer cell lines [19]. Thus, we hypothesized that targeting the cell‐surface pool of SorLA with a blocking antibody might disrupt its ability to support HER2/HER3 trafficking and thus restrain its oncogenic properties. We chose to investigate a monoclonal SorLA ab that targets the complement‐type repeat domains (CRD) in SorLA extracellular part (Fig. 3A) as we have established earlier that CRD contributes to the SorLA‐HER2 interaction [18]. We assessed its ability to inhibit the viability of SorLA silencing sensitive but intrinsically HER2 therapy‐resistant MDA‐MB‐361 cells [23]. In line with their resistant background, MDA‐MB‐361 cells did not respond to HER2‐targeting antibody trastuzumab (Fig. 3B). Treatment with SorLA ab alone did not exhibit a significant effect on MDA‐MB‐361 cell viability, indicating that antibody treatment does not have the same effect as depletion of the protein (Fig. 3B). However, combining trastuzumab with SorLA ab inhibited the viability of MDA‐MB‐361 cells (Fig. 3B), indicating that SorLA ab alters trastuzumab resistance *in vitro*. We did not observe such effect when SorLA ab was combined with lapatinib (Fig. S3A). Next, we used the CAM assay to validate the growth‐inhibitory effect of this SorLA ab and trastuzumab combination treatment *in ovo*. Consistent with the cell viability results *in vitro*, we observed that combining SorLA ab and trastuzumab inhibit MDA‐MB‐361 tumor growth *in ovo* (Fig. 3C). In addition, a similar inhibitory effect on tumor growth was observed with a 75% reduced dose of both antibodies (Fig. 3C). To elucidate the potential effects of SorLA ab or trastuzumab treatment alone or in combination on HER2‐regulated signaling, we assessed cyclin D1 levels, phosphorylation of HER2, and its downstream signaling to the protein kinase B (AKT) and ERK pathways in MDA‐MB‐361 cells [24]. Cyclin D1 levels, AKT, and ERK pathway activation were unchanged by any of the treatments (Fig. 3D and Fig. S3B‐D). However, trastuzumab as a single agent increased HER2 phosphorylation at two major autophosphorylation sites Tyr1248 and Tyr1221/1222, which couple HER2 to the Ras‐Raf‐MAP kinase pathway [25, 26], as well as Tyr1196 which together with Tyr1248 regulate HER2 stability [27]( Fig. 3D‐G). While SorLA ab alone did not exhibit any effect on these phosphorylation sites, combining SorLA ab with trastuzumab potentiated HER2 phosphorylation compared with trastuzumab treatment (Fig. 3D–G). This increase in tyrosine‐residue phosphorylation correlated with a decrease in total HER2 levels in trastuzumab‐ and the combination‐treated cells (Fig. 3D and H). Trastuzumab has been described to pose agonistic effects, resulting in increased HER2 phosphorylation and cell‐growth inhibition [28]. Therefore, these results suggest that HER2 phosphorylation might underpin the growth‐inhibitory effects of the combination treatment. Altogether, these results demonstrate that, even though SorLA antibody is not sufficient to inhibit cell viability alone, antibody‐based targeting of SorLA significantly alters resistance to HER2‐targeted therapy *in vitro* and *in ovo*, possibly through enhanced HER2 phosphorylation.

### 3.4 SorLA ab reduces resistance to HER2‐targeted therapy in a mouse xenograft model

**Fig. 4:**
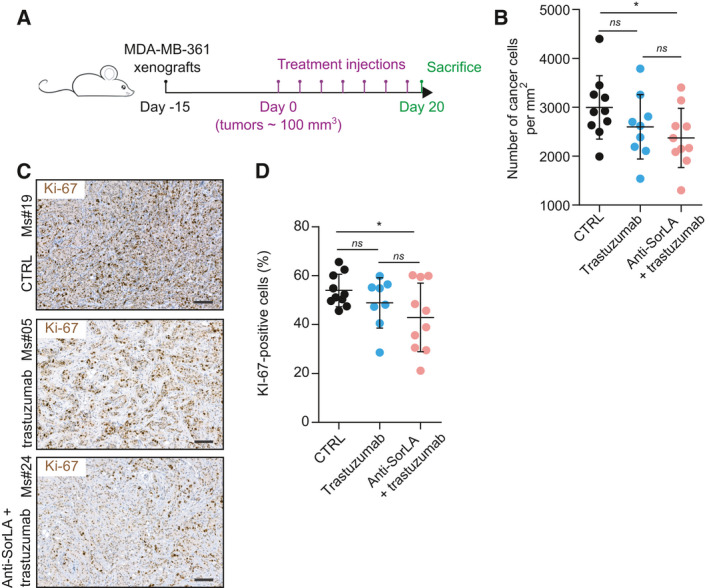
Anti‐SorLA and trastuzumab combination treatment exhibits anti‐tumor effects *in vivo*. (A) A scheme describing the timeline of the *in vivo* experiment. (B) Anti‐SorLA antibody and trastuzumab combination therapy decreases tumor cell density. Results are represented as mean ± SD, *n* = 9‐10 tumors/group. (C) Anti‐SorLA antibody and trastuzumab combination therapy decreases the number of Ki‐67‐positive cancer cells. Tumor sections from IgG control‐, trastuzumab‐, and anti‐SorLA with trastuzumab‐treated mice were stained for the proliferative marker Ki‐67. Scale bars: 100 µm. (D) Quantification of Ki‐67‐positive cancer cells in tumor sections described in (C). Results are represented as mean ± SD, *n* = 9‐10 tumors/group. Statistical analyses: *P < 0.05, Student’s *t*‐test (unpaired, two‐tailed, unequal variance).

To investigate the translational relevance of SorLA ab and trastuzumab combination treatment, we grafted MDA‐MB‐361 cells in Nude mice, which subsequently received either IgG control, trastuzumab, or SorLA ab and trastuzumab combination (Fig. 4A). Trastuzumab alone slightly inhibited the tumor growth of MDA‐MB‐361 cells (Fig. S4). This effect might have been due to the ability of trastuzumab to trigger a natural killer cell response [29, 30]. While tumor growth curves revealed no statistical difference between trastuzumab and the combination therapy (Fig. S4), further analysis of histopathological features revealed decreased tumor cell density and lower expression of the proliferative marker Ki‐67 in the tumors of mice treated with the combination therapy compared with trastuzumab‐treated and control mice (Fig. 4B–D). Altogether, these results indicate anti‐tumor activity for the SorLA ab and trastuzumab combination treatment *in vivo*.

### 3.5 SorLA ab alters cell proliferation specifically in HER2‐positive breast cancer patient‐derived explants

**Fig. 5:**
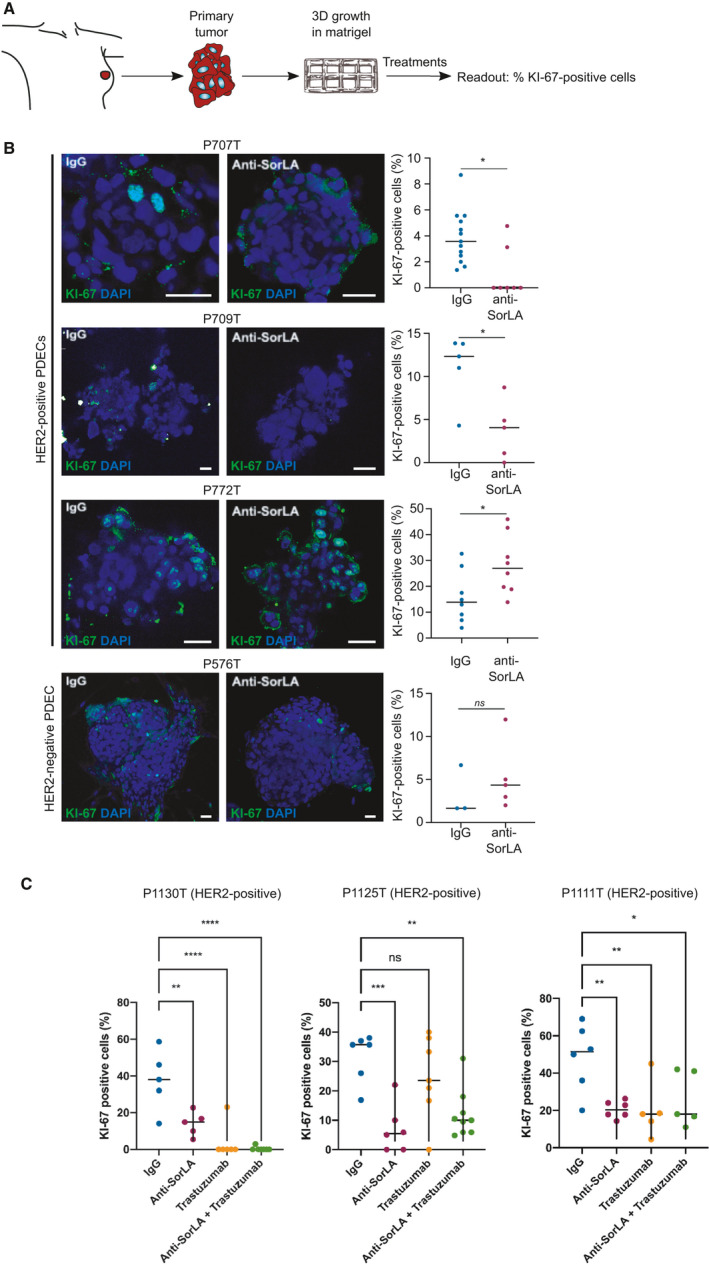
Anti‐SorLA antibody alters the proliferation of HER2‐positive patient tissue‐derived explant cultures. (A) Workflow for breast cancer patient tissue‐derived explant cultures (PDECs). PDECs were grown in Matrigel (see methods for details) and treated with either IgG control or anti‐SorLA antibody. Proliferation was assessed using Ki‐67 immunofluorescence staining. (B‐C) Anti‐SorLA inhibits the expression of the proliferation marker Ki‐67 in 5 out of 6 HER2‐positive PDECs. (B) PDECs from 3 HER2‐positive (P707T, P709T, and P772T) and 1 HER2‐negative (P576T) patients were treated with either IgG control or anti‐SorLA antibody (10 µg·mL^−1^) for 48 h prior to assessment of Ki‐67‐positive cells by confocal microscopy. Scale bars: 50 µm (C) PDECs from 3 HER2‐positive (P1130T, P1125T, and P1111T) were treated with either IgG control, anti‐SorLA antibody (10 µg·mL^−1^), trastuzumab (10 µg·mL^−1^), or both for 48 h prior to assessment of Ki‐67‐positive cells by confocal microscopy. SORLA and HER2 expression was analyzed from patient tumor samples corresponding to the PDEC with immunohistochemistry. Shown are representative images. Scale bar 10 μm. Each data point represents one explant; the bars indicate the average percentage of Ki‐67‐positive cells; statistical analyses: *P < 0.05; **P < 0.007; ***P < 0.001, ****P < 0.0001, C, Mann–Whitney *U*. D, one‐way ANOVA, Dunnett’s multiple comparisons test.

To determine whether SorLA ab shows anti‐tumor activity in authentic heterogeneous tumor tissue, we explored the effects of SorLA ab in patient‐derived explant cultures (PDECs). PDECs were derived fresh from breast cancer surgeries and grown in three‐dimensional matrix [31, 32]. IHC staining of the patient tumors corresponding to the PDEC revealed that the HER2‐positive tumors were also positive for SorLA expression, while HER2‐negative tumor expressed low SorLA levels (Fig. S5). Primary PDECs were treated with SorLA ab or IgG control and subsequently assessed for the expression of the proliferation marker Ki‐67 (Fig. 5A). We found that SorLA ab alone strongly inhibited Ki‐67 expression in 2 out of 3 HER2‐positive PDECs (Fig. 5B). Importantly, SorLA ab did not alter the proliferation of PDECs from HER2‐negative breast cancer (Fig. 5B), suggesting specific anti‐cancer effects for SorLA ab in HER2‐positive breast cancer. Next, we tested whether combining anti‐SorLA with trastuzumab would further augment the antiproliferative effect. In all of these HER2‐ and SORLA‐positive PDECs (Fig. 5C), anti‐SorLA monotherapy provided a strong and significant reduction in the percentage of Ki‐67‐positive cells. Sample P1130T was very sensitive to trastuzumab, and in this specimen, anti‐SorLA alone was 10% less potent. In P1125T and P1111T PDEC cultures, anti‐SorLA was as efficient or more efficient than trastuzumab alone and there was no added benefit from the combination (Fig. 5C). These results demonstrate that anti‐SorLA monoclonal antibody shows activity in a clinically relevant model of HER2‐positive breast cancer.

## 4 Discussion

Here, we provide for the first time proof‐of‐concept evidence supporting the druggability of SorLA in cancer. A commercially available SorLA ab synergizes altered resistance to HER2 monoclonal antibody trastuzumab *in vitro*, *in ovo*, and in Nude mouse xenografts *in viv*o. Furthermore, SorLA ab alone exhibited anti‐cancer effects specifically in HER2‐positive PDECs.

The SorLA extracellular region is a mosaic structure comprising multiple subdomains shown to mediate ligand binding/discharge. SorLA ab targets the CRD known to be, along with the VPS10P domain, a major site for ligand recognition [33, 34] and involved in the direct interaction between HER2/HER3 and SorLA [18, 19]. We have previously shown that both the extracellular domain of SorLA and its C‐terminal tail were necessary for HER2 binding and trafficking, respectively [18]. We anticipate that SorLA ab triggers its anti‐tumor effects upon binding to the cell‐surface pool of SorLA prior to the retrograde trafficking pathway since it is reported that low pH, corresponding to acidic milieu of endocytic vesicles, decreases CRD binding to cargo proteins [35]. Thus, we anticipate possible resistance mechanisms to this treatment through altered subcellular traffic of SorLA, which may deviate the receptor from the cell surface of treatment‐resistant cancer cells.

In our *in vivo* mouse experiment, we used MDA‐MB‐361 cells that originate from brain metastatic HER2‐positive breast cancer and represent a well‐established model of primary HER2 therapy resistance [23, 36]. Our *in vivo* results showed that the combination treatment, but not trastuzumab alone, decreased tumor cell density and Ki‐67 expression, with no difference between tumor growth curves from the combination treatment vs trastuzumab. This suggests a delayed action of SorLA ab *in vivo*. A future study could assess the effect of the combination treatment on tumor growth with extended treatment administration. Importantly, we did not observe any signs of toxicity such as weight loss and behavioral changes in mice receiving SorLA ab and trastuzumab combination treatment, albeit the SorLA ab used is reactive against mouse SorLA. This indicates cancer specificity of the treatment most likely owing to the high SorLA overexpression specific for HER2‐positive breast cancer cells [18]. Furthermore, our CAM assay data show similar anti‐tumor effects between a full and a reduced dose of SorLA ab and trastuzumab combination treatment suggesting that even lower doses of SorLA ab treatment with reduced risk of adverse effects may reach therapeutic efficacy *in vivo*.

Individual SorLA ab treatment was able to inhibit the proliferation in HER2‐positive PDECs. These results in a clinically relevant *ex vivo* model, shown to recapitulate the complex tumor heterogeneity [31, 37], are promising in terms of translational potential. Interestingly, SorLA ab did not exhibit any significant effects in the HER2‐negative PDECs. This result is in line with our previous study demonstrating that SorLA promotes oncogenicity specifically in HER2‐driven cancers [18]. Given that a) endosomal localization of HER2 correlates with therapy resistance [38] and b) SorLA mediates HER2 endosomal trafficking [18], determining co‐localization between SorLA and HER2 might have prognostic value in HER2‐positive breast cancer.

## 5 Conclusions

SorLA promotes bladder cancer growth by sustaining HER2 signaling [18]. The efficacy of SorLA ab in inhibiting the progression of SorLA‐dependent HER2‐driven cancers, other than breast cancer, will need to be assessed in future studies. Furthermore, SorLA overexpression in adipocytes enhances obesity by bolstering insulin receptor signaling through increased receptor recycling [16]. Therefore, targeting SorLA in cancer might be translatable to other SorLA‐promoted human diseases.

## Conflict of interest

The authors declare no conflict of interest.

### Peer Review

The peer review history for this article is available at https://publons.com/publon/10.1002/1878‐0261.13106.

## Author contribution

HA, MP, JK, and JI conceptualized the data; HA, MP, JL, EP, JMA, HMH, PMM, JK, and JI involved in methodology; HA, JL, EP, and PMM made formal analysis. HA, MP, JL, EP, JMA, HMH, and PMM investigated the study; HA and JI involved in writing—original draft; HA, JL, EP, PMM, JK, and JI involved in writing—review and editing; JK and JI supervised the study; and HA, JK, and JI acquired the funding.

## Supporting information


**Table S1**. Information related to the primary antibodies used in this study.
**Fig. S1**. Lapatinib and trastuzumab effects on SorLA expression.
**Fig. S2**. SorLA silencing inhibits cell viability and colony growth in vitro and tumor growth in ovo.
**Fig. S3**. Drug and antibody effects on cell viability and signaling.
**Fig. S4**. Trastuzumab and anti‐SorLA effects on tumor growth.
**Fig. S5**. Immunohistochemical staining of SorLA and HER2 from tumors corresponding to the PDECs.

## Data Availability

The authors declare that the data supporting the findings of this study are available within the article and from the authors on request.
