# Supplementary material for: Sortilin‐related receptor is a druggable therapeutic target in breast cancer
Source: Mol Oncol. 2021 Oct 10;16(1):116–29. doi: 10.1002/1878-0261.13106 (PMC8732349; doi:10.1002/1878-0261.13106)
Supplement: Supplementary file 1 — Table S1. Information related to the primary antibodies used in this study. Fig. S1. Lapatinib and trastuzumab effects on SorLA expression. Fig. S2. SorLA silencing inhibits cell viability and colony growth in vitro and tumor growth in ovo. Fig. S3. Drug and antibody effects on cell viability and signaling. Fig. S4. Trastuzumab and anti‐SorLA effects on tumor growth. Fig. S5. Immunohistochemical staining of SorLA and HER2 from tumors corresponding to the PDECs. [file MOL2-16-116-s001.docx]

Table 1: information related to the primary antibodies used in this study.

| **Antibody** | **Manufacturer** | **Catalogue No.** | **Application**  **(concentration)** |
| --- | --- | --- | --- |
| Phospho-ERK1/2 (Thr202/Tyr204) | Cell Signaling Technology | #4370S | WB (1:1000) |
| ERK1/2 | Cell Signaling Technology | 9102S | WB (1:1000) |
| LR11 (SORL1) | BD Transduction Lab | 612633 | WB (1:1000) |
| SORL1 | Atlas Antibodies | HPA031321 | IHC (1:300) |
| α-tubulin | Hybridoma Bank | 12g10 | WB (1:4000) |
| Ki-67 | Abcam | ab15580 | IF (1:300) |
| Ki-67 | Millipore | #AB9260 | IHC (1:1000) |
| Cyclin D1 | Cell Signaling Technology | E3P5S | WB (1:1000) |
| Phospho-HER2 (Tyr1196) | Cell Signaling Technology | 6942S | WB (1:1000) |
| Phospho-HER2 (Tyr1221/1222) | Cell Signaling Technology | 2243T | WB (1:1000) |
| Phospho-HER2 (Tyr1248) | Cell Signaling Technology | 2247S | WB (1:1000) |
| HER2 (e2-4001 + 3B5) | Thermo Scientific | MA5-14057 | WB (1:1000) |
| Phospho-AKT (S473) | Cell Signaling Technology | 9271S | WB (1:1000) |
| AKT | Cell Signaling Technology | 9272S | WB (1:1000) |


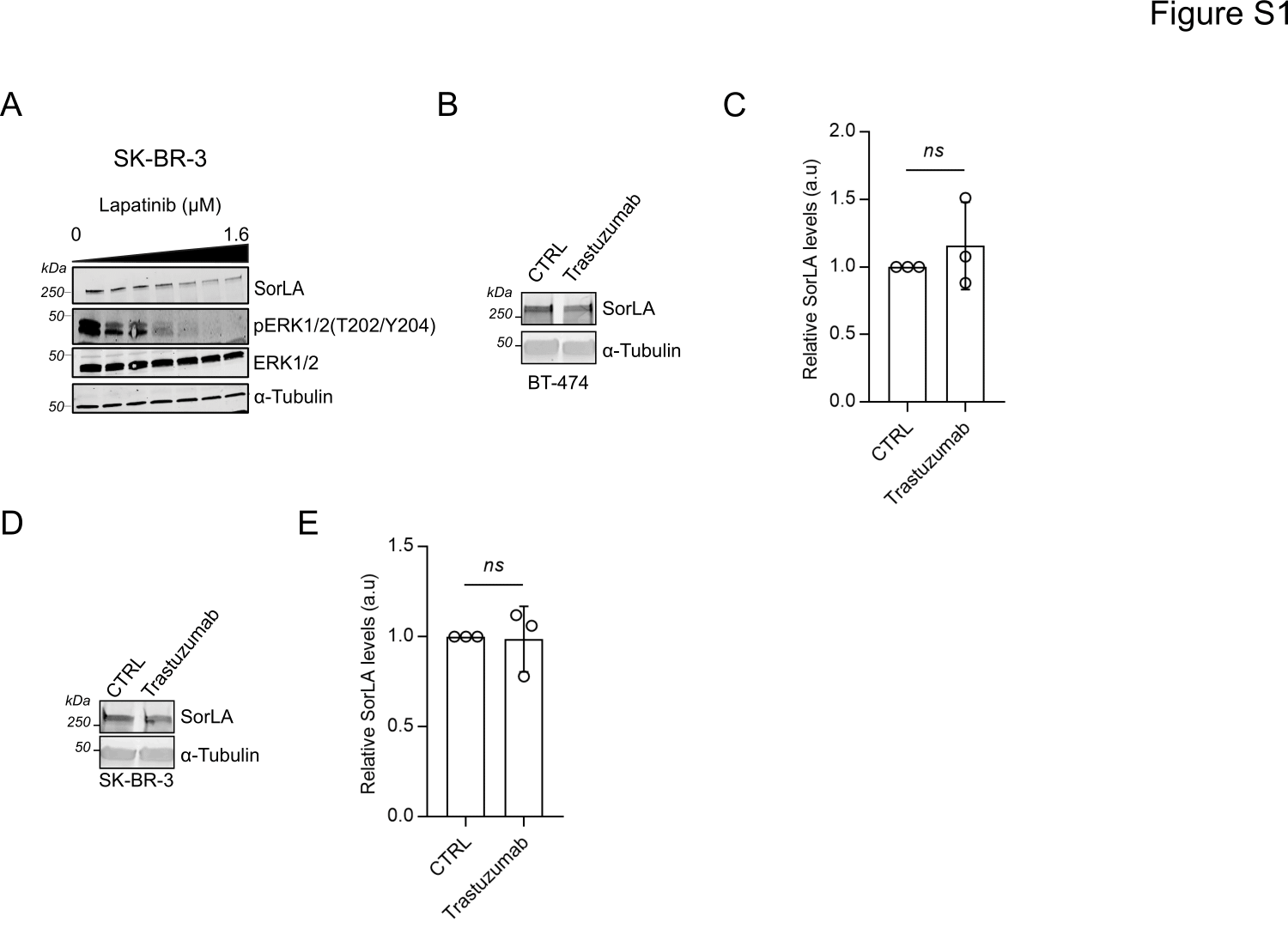


**Figure S1 related to Figure 1. Lapatinib and trastuzumab effects on SorLA expression**

1. Lapatinib decreases SorLA levels. SK-BR-3 cells were treated with increasing concentrations of lapatinib (0, 0.1, 0.2, 0.4, 0.8, and 1.6 µM) for 24 h. Representative immunoblotting of SorLA, pERK1/2(T202/Y204), and total ERK1/2, with α-tubulin as a loading control.
2. Western blot analysis of SorLA expression in trastuzumab-treated (10 µg/mL; 24 h) BT-474 cells with α-tubulin as a loading control.
3. Band intensity quantification of SorLA levels in (B) normalized to the loading control and relative to IgG-treated control sample.
4. Western blot analysis of SorLA expression in trastuzumab-treated (10 µg/mL; 24 h) SK-BR-3 cells with α-tubulin as a loading control.
5. Band intensity quantification of SorLA levels in (D) normalized to the loading control and relative to IgG-treated control sample.

Results are represented as mean ± SD. Statistical analyses: Student’s t-test (unpaired, two-tailed, unequal variance).


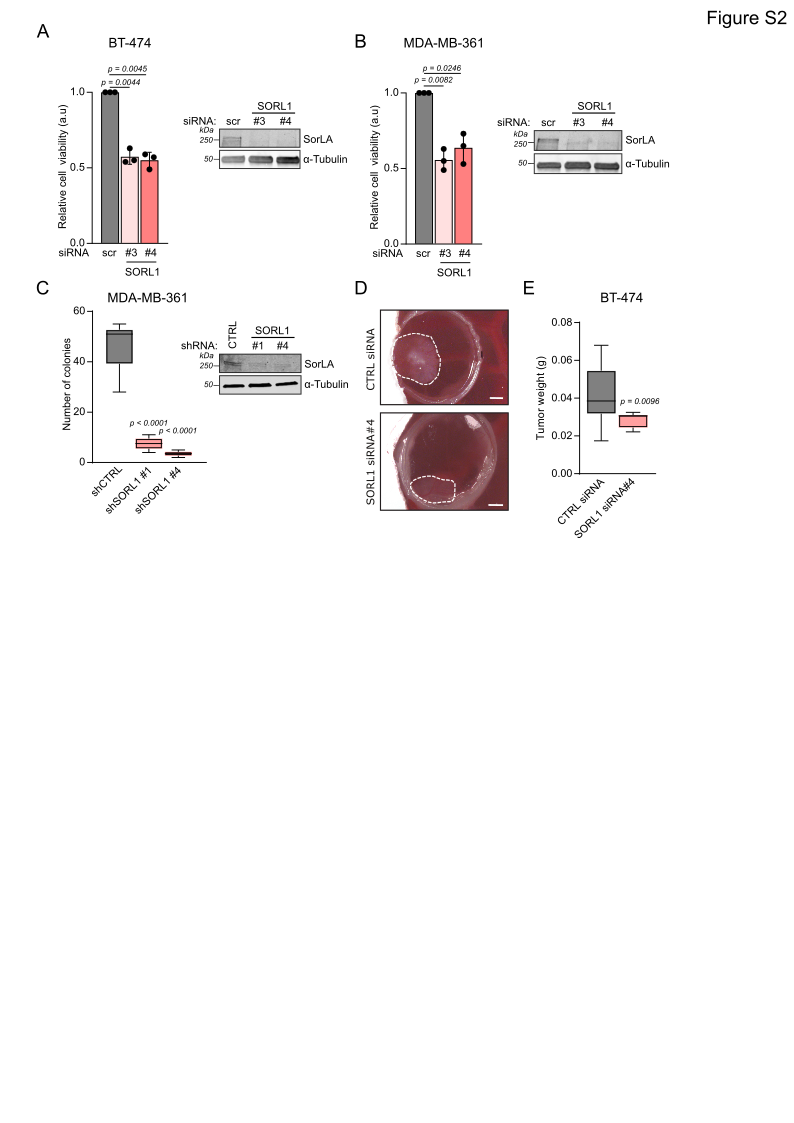


**Figure S2 related to Figure 2. SorLA silencing inhibits cell viability and colony growth *in vitro* and tumor growth *in ovo*.**

1. BT-474 cells were transiently silenced for SorLA expression and cell viability was assessed using the WST-8-based method. Each data point represents the average of 3 internal replicates. Results are represented as mean ± SD. A representative western blot validating SorLA silencing is shown.
2. MDA-MB-361 cells were transiently silenced for SorLA expression and cell viability was assessed using the WST8-based method. Each data point represents the average of 3 internal replicates. Results are represented as mean ± SD. A representative western blot validating SorLA silencing is shown.
3. SorLA silencing inhibits colony formation. Colony formation assay using MDA-MB-361 cells stably expressing CTRL shRNA, SORL1 ShRNA#1 and ShRNA#4. Results are represented as mean ± SEM. A representative western blot validating SorLA silencing is shown.
4. SorLA silencing inhibits *in ovo* tumor growth. *In ovo* CAM tumor formation assay with SorLA-silenced BT-474 cells. Scale bars: 1 cm.
5. Tumors described in (D) were weighed and the results are represented as median ± min to max. N ≥ 8 tumors / group.

Statistical analyses: Student’s t-test (unpaired, two-tailed, unequal variance).


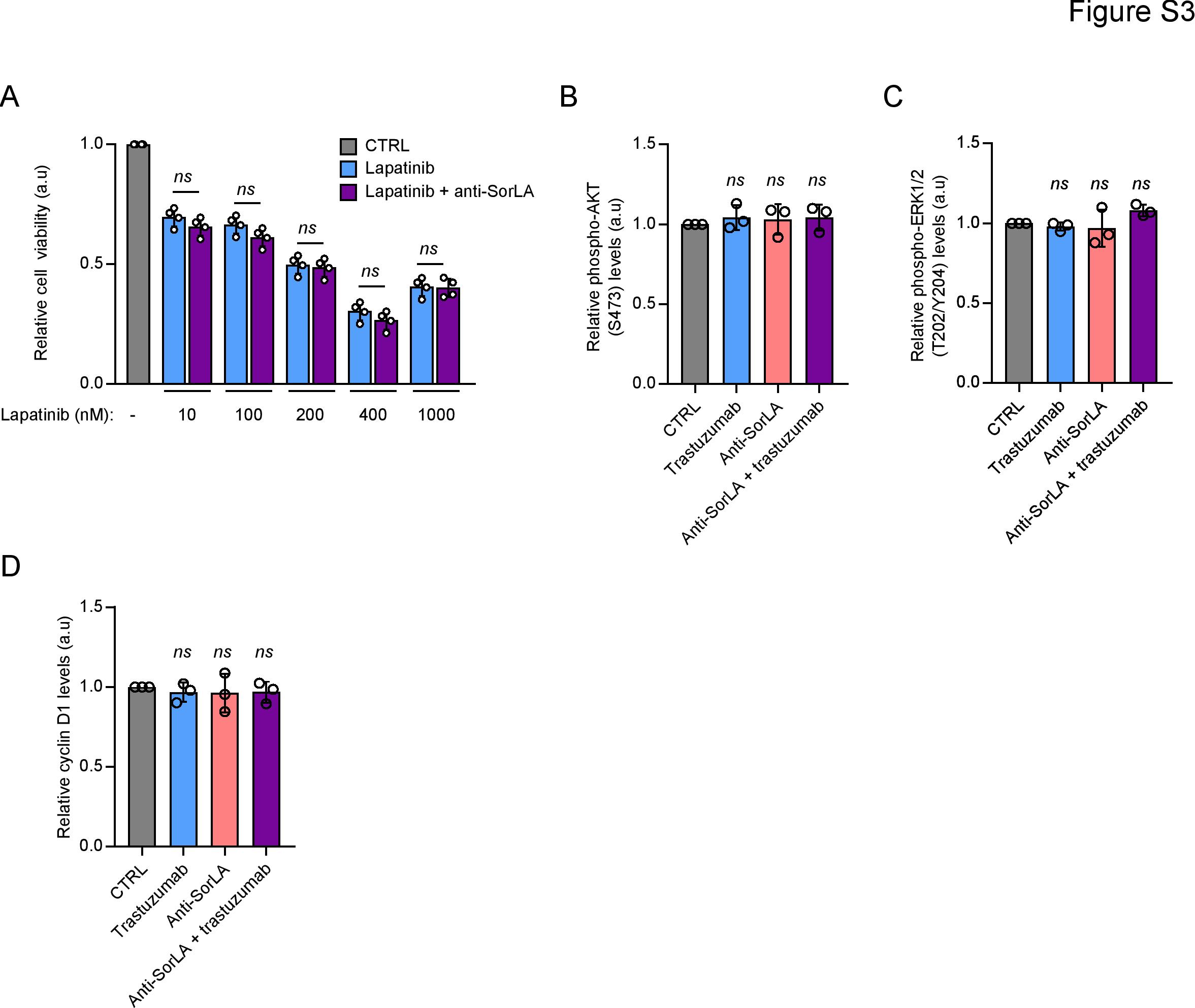


**Figure S3 related to Figure 3. Drug and antibody effects on cell viability and signaling.**

1. Cell viability assay of MDA-MB-361 cells treated with serial concentrations of lapatinib alone or in combination with anti-SorLA antibody (20 µg/mL) for 48 h.
2. Band intensity quantification of phosphorylated AKT (S473; Figure 3D), normalized to total AKT and relative to IgG-treated control sample.
3. Band intensity quantification of phosphorylated ERK1/2 (T202/Y204; Figure 3D), normalized to total ERK1/2 and relative to IgG-treated control sample.
4. Band intensity quantification of cyclin D1 (Figure 3D), normalized to loading control and relative to IgG-treated control sample.

(A-D) Results are represented as mean ± SD. Statistical analyses: Student’s t-test (unpaired, two-tailed, unequal variance).


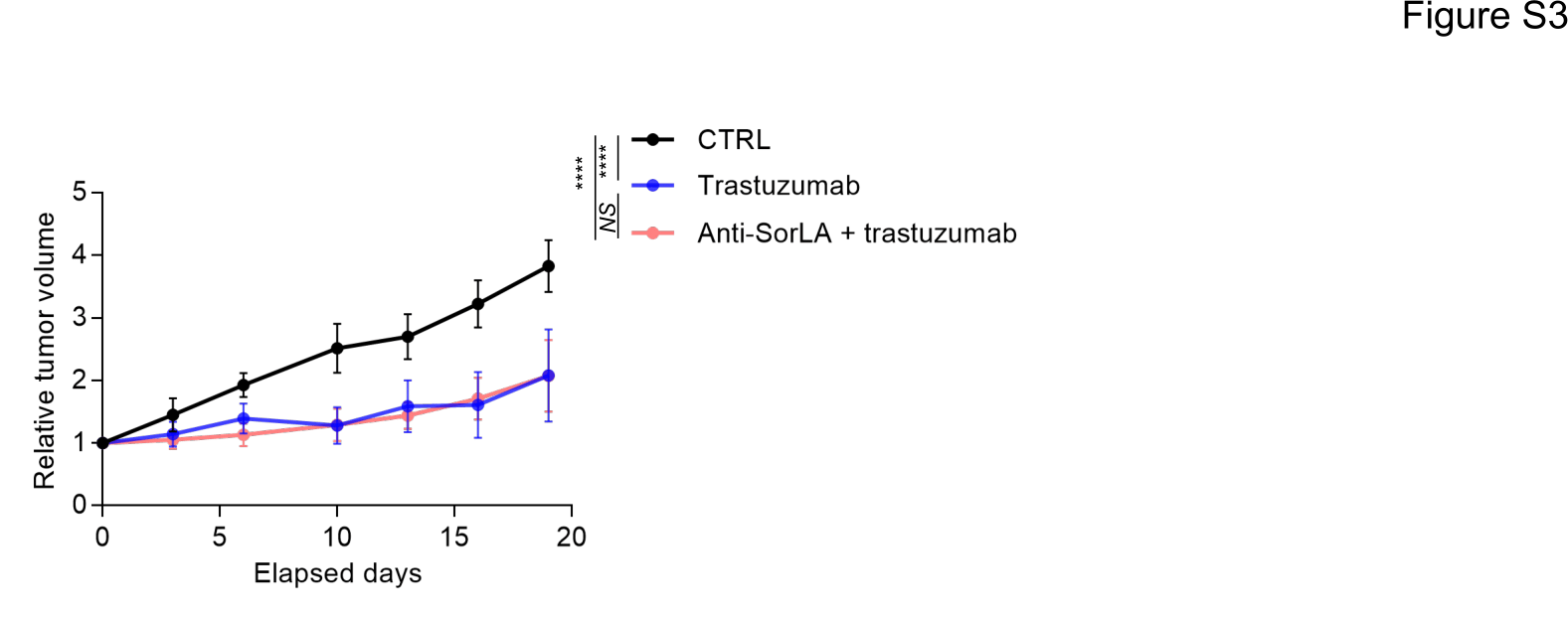


**Figure S4 related to Figure 4. Trastuzumab and anti-SorLA effects on tumor growth.**

Tumor growth curves from IgG control-, trastuzumab-, and anti-SorLA with trastuzumab-treated mice. Results are represented as mean relative to day 0 ± SEM. Statistical analyses: exponential growth curve fit comparisons; extra sum-of-squares F test. *****P<0.0001.*


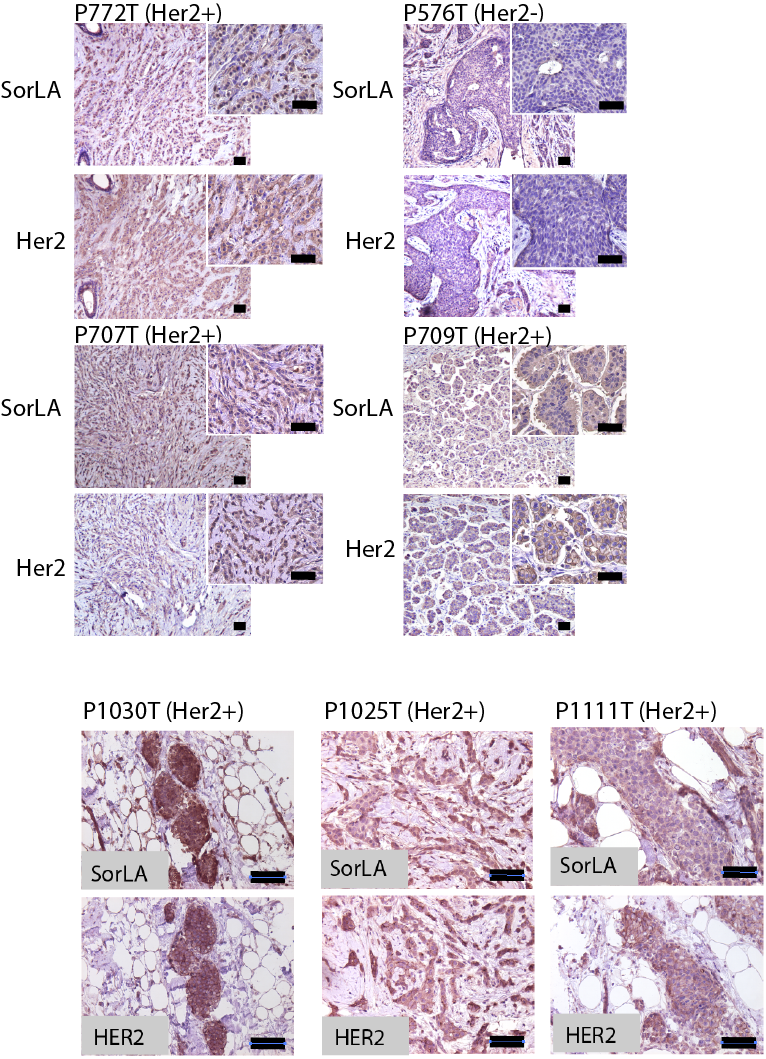


**Figure S5 related to Figure 5**: **Immunohistochemical staining of SorLA and HER2 from tumors corresponding to the PDECs.** SorLA and HER2 were stained from tumor sections corresponding to HER2-positive (P772T; P707; P709T, P1130T, P1025T and P1111T) and one HER2-negative (P576T) patient-derived explant cultures. Scale bar 50 μm.
